# Insulin Increases Sestrin 2 Content by Reducing Its Degradation through the PI_**3**_K/mTOR Signaling Pathway

**DOI:** 10.1155/2015/505849

**Published:** 2015-02-22

**Authors:** Dandan Chai, Guoyu Wang, Ziyu Zhou, Hanyan Yang, Zhiwen Yu

**Affiliations:** ^1^Fujian Key Laboratory of Chinese Materia Medica, Biomedical Drug Research and Development Center, Fujian University of Traditional Chinese Medicine, Fuzhou 350108, China; ^2^Guangdong Provincial Key Laboratory of Food, Nutrition and Health, Department of Nutrition, School of Public Health, Sun Yat-Sen University, Guangzhou 510080, China

## Abstract

Sestrin (SESN) is known as a cysteine sulfinic acid reductase. Recently, nonredox functions of SESN in metabolic regulation and antitumor property have been recognized. While mechanisms underlying the expression of SESN are not fully understood. Here we report that insulin markedly increased SESN2 level in HepG2 cells through mTOR activation. To determine whether insulin affects SESN2 degradation, we assessed SESN2 turnover by applying the protein synthesis inhibitor, cycloheximide (CHX), and found that following insulin treatment SESN2 protein levels were reduced significantly slower than non-insulin-treated cells. Furthermore, the proteasomal inhibitor, MG132, dramatically increased SESN2 protein and its ubiquitination level while in the presence of MG132 insulin did not further increase SESN2 content, suggesting that insulin increases SESN2 content mainly via inhibiting its proteasomal degradation. We then explored the potential feedback role of SESN2 in insulin signaling by SESN2 siRNA knockdown in HepG2 cells. Following SESN2 knockdown insulin-stimulated PKB phosphorylation was enhanced and accompanied by reduced PTEN content. Taken together, our study suggests that insulin upregulates SESN2 content via the PI_3_K/mTOR signaling pathway and this effect is attributed to decreased SESN2 degradation. Furthermore, SESN2 via modulating PTEN plays a negative feedback role in insulin signaling.

## 1. Introduction

Sestrins (SESNs) belong to a highly conserved protein family composed of three members in mammals, that is, SESN1, SESN2, and SESN3, while there is only one SESN gene identified in lower organisms, such as* Drosophila melanogaster* and* Caenorhabditis elegans* [1]. Initial investigations on SESNs function have recognized its role in regulating redox balance by repairing oxidized peroxiredoxin against oxidative stress [2, 3]. In addition to the redox-regulating function, the inhibitory effect of SESNs on tumor pathological processes including cell transformation and genomic instability has been reported [4–6]. Several signaling molecules important for the modulation of tumor growth can be affected by SESNs. AMPK by regulating glucose metabolism, lipid oxidation, and protein synthesis plays antitumor function [7]. Overactivated mTOR function is often observed in a variety of tumors promoting protein synthesis and tumor growth [8]. In contrast, SESNs by interacting with AMPK play an inhibitory effect on mTOR function [7]. Insulin is one of important regulators promoting anabolism by activating mTOR and recent studies suggest that SESN could crosstalk with insulin signaling. Insulin signaling-mediated mTOR activation has been shown to upregulate dSESN expression in* Drosophila* [1]. In addition, the ablation of SESNs has been shown to exacerbate obesity-induced mTORC1/S6K activation in mice [9].

Among the three SESN family members, SESN2 has been studied most and was reported to be regulated by the tumor suppressor p53 via promoting transcriptional expression of SESN2 [5, 10]. As a stress-responsive protein, SESN2 expression was stimulated by various stresses including oxidative, genotoxic, and energetic stress stimuli [4, 5]. Both oxidative stress and genotoxic stimuli can activate SESN2 in a p53-dependent manner [11, 12]. However, a study by Ben-Sahra et al., in PC3 p53 null cells, has shown that energetic stress induces SESN2 expression, suggesting a p53-independent mechanism [13]. dSESN expression can be stimulated by activating insulin/dTOR-mediated production of reactive oxygen species (ROS) [1]. In addition, in high-fat-diet-fed mice both mTOR overactivation and elevated SESN2 expression were evident [9]; we proposed that that hyperinsulinemia-evoked mTOR activation might upregulate SESN2 protein level. We, therefore, in the present study examined whether in mammalian cells insulin could directly regulate SESN2 expression. Furthermore, we investigated mechanisms involved in SESN2 expression and the functional impact of this regulation on insulin signaling.

We initiated our investigation in a human hepatic cell line and in mouse primary hepatic cells in culture. We found that insulin through mTOR activation significantly increased SESN2 protein content in both HepG2 cells and primary mouse hepatocytes. This increase is likely due to the inhibition of SESN2 degradation in proteasome. In addition, we showed that SESN2 siRNA knockdown enhanced insulin-stimulated PKB phosphorylation by reducing PTEN expression that can be a novel signaling loop for its regulation on insulin signaling.

## 2. Materials and Methods

### 2.1. Materials

The human hepatic carcinoma cell line HepG2 was obtained from American Type Culture Collection (ATCC, Manassas, USA). The PKB inhibitor triciribine (TCN), proteasomal inhibitor MG132, lysosomal inhibitor chloroquine (CQ), protein synthesis inhibitor cycloheximide, PI_3_K inhibitor LY294002, JNK inhibitor SP600125, p38 inhibitor SB202190, and ERK inhibitor U0126 were all purchased from ENZO Life Sciences (Farmingdale, USA). Glucose-free Dulbicco's Modified Eagle's medium (DMEM) was obtained from Sigma-Aldrich (Saint Louis, USA). Insulin was purchased from Novo Nordisk (Clayton, USA). DMEM containing 4500 mg/L high glucose, D-Hanks buffer, Hanks' Balanced Salt Solution, RPMI 1640 medium, fetal bovine serum, Opti-MEM, penicillin-streptomycin, and trypsin-EDTA were obtained from GIBCO (Carlsbad, USA). Collagenase was from Worthington Biochemicals (Freehold, USA). Antibodies against PKB, PKB Ser473 and Thr308, S6K, p-S6K (Ser235/236), and rapamycin were purchased from Cell Signaling Technology (Danvers, USA). *β*-actin and SESN2 antibodies were purchased from Proteintech Group (Chicago, USA). Antibody against polyubiquitin was purchased from Biomo (Hornby, ON, Canada). Phosphatase and tensin homologue deleted on chromosome ten (PTEN) and horseradish peroxidase- (HRP-) conjugated secondary antibodies, protein A/G-Agarose beads, the human SESN2 siRNA, and the control siRNA were purchased from Santa Cruz (Santa Cruz, USA). Lipofectamine 2000 was from Invitrogen (Auckland, New Zealand). The enhanced chemiluminescence (ECL) reagents were from Thermo Fisher (Rockford, USA).

### 2.2. Cell Culture

HepG2 cells were cultured in DMEM (high glucose) supplemented with 10% fetal bovine serum, 100 units/mL penicillin, and 100 *μ*g/mL streptomycin. Cells were maintained at 37°C with humidified air and 5% CO_2_. Before experiments, the cells were serum-starved for 12 h and then treated with indicated reagents.

Mouse primary hepatic cell isolation and culture were performed according to the reported with some modifications [14]. Briefly, newborn C57BL/6J mice were decapitated and the liver was promptly taken. After washing in D-Hanks buffer to remove any blood, the liver tissue was minced into 3 to 4 mm^3^ pieces with sterile scissors. After washing the tissue pieces for 3 times, Hanks' Balanced Salt Solution containing 100 U/mL collagenase was added and the tissue was digested at 37°C for 2 h in a water bath shaking at 100 RPM. The cell suspension was filtrated through a sterile stainless steel mesh to separate the dispersed cells and tissue fragments. The separated cells were washed with RPMI 1640 medium and centrifuged at 800 g for four times. The cells were then cultured in RPMI 1640 medium containing 10% fetal bovine serum, 100 units/mL penicillin, and 100 *μ*g/mL streptomycin.

### 2.3. Immunoprecipitation and Western Blot Analysis

Cultured cells were collected and lysed with the buffer containing phosphatase and protease inhibitors as described previously [15]. Equal amount of proteins was used for immunoprecipitation or western blot analysis. For SESN2 immunoprecipitation, 1 mg cellular protein was precleared by incubation with protein A/G-Agarose beads in 20% (w/v) suspension for 2 h at 4°C on a rotator. The mixture was then centrifuged at 13,400 g for 20 min at 4°C. The supernatant was collected and 1 *μ*g anti-SESN2 antibody was added. After incubation overnight at 4°C, 20 *μ*L protein A/G-Agarose beads were added for 2 h at 4°C. The immunocomplex was collected by centrifugation and washed three times with HEPES buffer (50 mM, pH 7.4) containing 1 mM EDTA, 150 mM NaCl, 1% NP-40, 0.5% sodium deoxycholate, 0.1% SDS, 1 *μ*M phenylmethanesulfonyl fluoride (PMSF), and 5 *μ*M MG132. The SESN2 immunoprecipitates or total cell lysates were dissolved in sodium dodecyl sulfate (SDS) sample loading buffer and heated at 95°C for 5 min. Proteins were separated by 10% SDS-PAGE and then transferred to nitrocellulose filter membranes. After blocking, the membranes were probed with specific primary antibodies at 4°C overnight and then incubated with appropriate HRP-conjugated secondary antibodies. Protein bands were visualized with ECL. The levels of protein expression were examined by densitometry with Quantity One software, as previously described [16].

### 2.4. siRNA Transfection

For transient siRNA transfection, HepG2 cells were seeded on 6-well plates in antibiotic-free DMEM. When the cells were grown into 30–50% confluence, SESN2 siRNA or control siRNA was transfected using Lipofectamine 2000, following the manufacturer's protocol. After 2 or 3 days, the cells were collected and lysed for western blot analysis.

### 2.5. Statistical Analysis

Data are expressed as the mean value ± SEM. Statistical comparisons of mean among different groups were carried out by using either Student's *t*-test or one-way ANOVA when appropriate and *P* value <0.05 was considered as statistically significant.

## 3. Results

### 3.1. Insulin Dramatically Increases SESN2 Protein Content in Hepatic Cells

**Figure 1:**
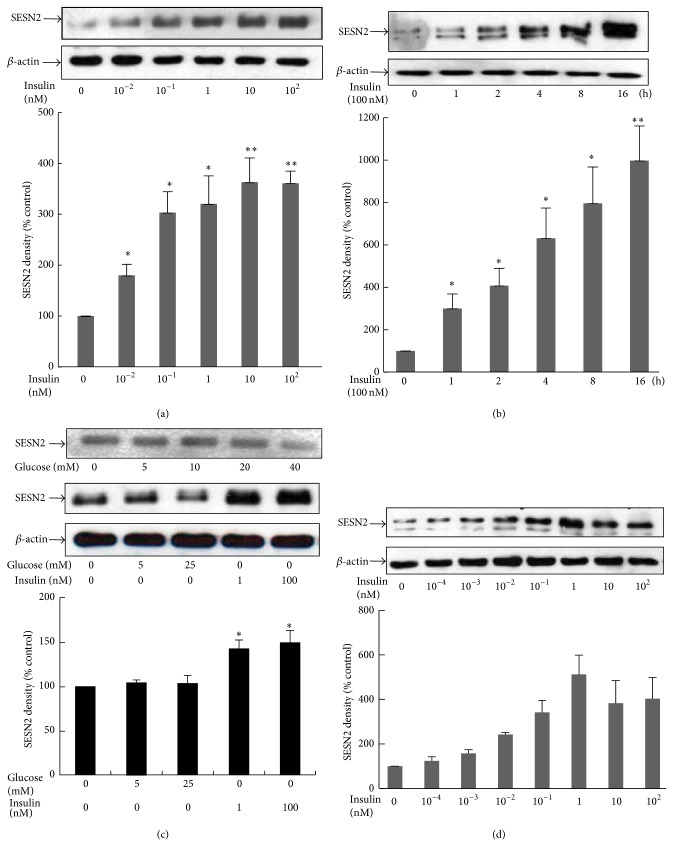
Insulin treatment upregulates SESN2 protein levels in hepatic cells. HepG2 cells were incubated with indicated concentrations of insulin for 18 h (a and c) or 100 nM insulin for indicated time course (b). Cells were harvested for western blotting. In (a) and (b), HepG2 cells were cultured in DMEM containing 25 mM glucose. In (c), cells were grown with the indicated concentrations of glucose. (d) Primary mouse hepatic cells were treated with the indicated concentrations of insulin for 18 h, followed by cell harvesting and western blotting. Upper panels: western blot of SESN2 and *β*-actin (loading control); lower panel: densitometric analysis of two independent experiments. ^*^ *P* < 0.05; ^**^ *P* < 0.01 compared with noninsulin treatment.

The upregulating effect of insulin signaling on dSESN expression reported in a low organism, that is,* Drosophila* [1], prompted us to investigate whether insulin could also modulate SESN2 expression in mammalian cells. Our investigation was started with HepG2 cells, grown with DMEM containing 25 mM glucose or indicated concentrations of glucose in serum-free condition and treated with different concentrations of insulin for 18 h, followed by examining SESN2 levels by western blotting. As shown in Figure 1(a), an 18 h exposure to insulin concentration dependently increased SESN2 levels. The stimulatory effect of insulin on SESN2 expression was evident at 0.01 nM (almost 2-fold) and reached the maximal effect at the concentrations of 10–100 nM (about 3.5-fold compared to insulin-untreated control). We have also examined the effect of insulin on SESN2 expression with a 1–16 h time course. As shown in Figure 1(b), the treatment of HepG2 cells with 100 nM insulin rapidly increased SESN2 level at 1 h (about 3-fold increase) and the stimulatory effect was more significant thereafter. We have also observed that when HepG2 cells were grown in glucose-free medium, insulin was still able to induce a significant increase in SESN2 level (Figure 1(c)). We then performed similar experiments in primary mouse hepatic cells. As shown in Figure 1(d), insulin concentration dependently increased SESN2 content. The maximum effect was observed when insulin reached 1 nM concentration. These observations indicate for the first time that SESN2 level in mammalian hepatocytes can be upregulated by physiological concentrations of insulin.

### 3.2. Insulin-Stimulated SESN2 Expression Is Dependent on the PI_3_K/PKB/mTOR Signaling Pathway

**Figure 2:**
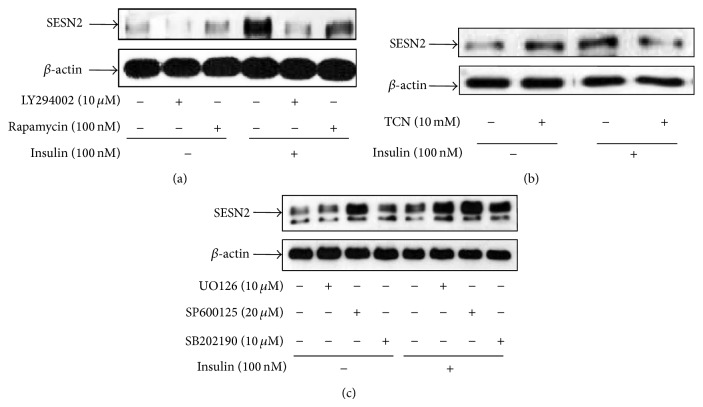
PI_3_K/mTOR signaling pathway is required for the upregulation of SESN2 level by insulin treatment. HepG2 cells were pretreated with indicated reagents at the indicated concentrations for 1 h, followed by an 18 h further treatment with 100 nM of insulin. Cells were harvested for western blotting. (a) to (c) are representative images of at least three independent experiments.

To identify the potential signaling pathways involved in insulin-stimulated SESN2 expression, HepG2 cells were pretreated with the PI_3_K inhibitor LY294002, the PKB inhibitor triciribine (TCN), and the mTOR inhibitor rapamycin, respectively, for 1 h followed by 100 nM insulin treatment for 18 h. All the three inhibitors either completely blocked or significantly attenuated insulin-stimulated SESN2 levels (Figures 2(a) and 2(b)). Insulin can also activate the MAP kinase signaling pathways including JNK, p38, and ERK [17]. In addition, it has been shown that JNK-activated dFoxO mediates dSESN upregulation in* Drosophila* [1]. We therefore examined the effects of the ERK inhibitor U0126, the p38 inhibitor SB202190, and the JNK inhibitor SP600125 on SESN2 levels in HepG2 cells and observed that none of them generated any appreciable inhibitory effect on insulin-stimulated SESN2 levels (Figure 2(c)). Together, these data suggest that insulin-regulated SESN2 expression is through the activation of PI_3_K/PKB/mTOR signaling but not the MAP kinase-mediated signaling.

### 3.3. Insulin and Proteasomal Inhibitor Block SESN2 Degradation

**Figure 3:**
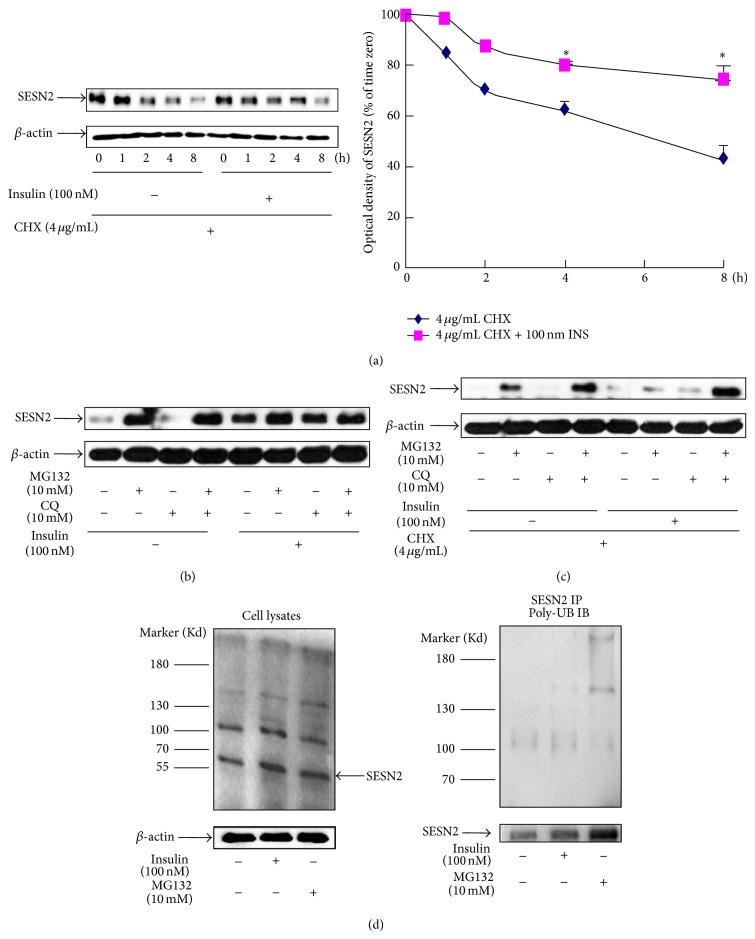
Insulin or the proteasomal inhibitor MG132 blocks SESN2 degradation. (a) HepG2 cells were pretreated with CHX for 30 min followed by a treatment with or without 100 nM of insulin (INS) for the indicated times. The cells were then lysed for SESN2 western blotting. Right panel is the plot of densitometric analysis. ^*^ *P* < 0.05 compared with noninsulin treatment. (b) and (c) HepG2 cells were treated with the indicated reagents for 30 min, followed by a treatment with or without 100 nM insulin for 8 h. The cells were then lysed for SESN2 western blotting. (d) HepG2 cells were incubated with or without insulin or MG132 for 18 h and the cells were lysed. Western blotting of SESN2 and *β*-actin (loading control) of total cellular proteins (left panel) or SESN2 immunoprecipitation followed by polyubiquitin antibody western blotting (right panel). IP indicates the immunoprecipitation antibody; IB indicates the immunoblotting antibody. Bottom panel of left panel: western blotting of the input samples. A representative blot of three independent experiments.

Since insulin treatment rapidly upregulated hepatic SESN2 protein within 1 h, it is unlikely that the stimulation is via the increase of SESN2 protein synthesis. We hence explored whether insulin-stimulated SESN2 expression is due to the blockage of its degradation. For this purpose, HepG2 cells were pretreated with the protein synthesis inhibitor cycloheximide (CHX) to prevent the production of newly synthesized SESN2, before insulin treatment. We observed that, in the presence of insulin, SESN2 disappeared slower than non-insulin-treated cells (Figure 3(a)), indicating that insulin may block or attenuate SESN2 degradation. To further identify which degradation pathway is involved in SESN2 degradation, we examined the potential role of two major cellular protein-degrading systems, that is, proteasome and lysosome on SESN2 turnover. HepG2 cells were pretreated with either the proteasomal inhibitor, MG132, or the lysosomal inhibitor, chloroquine (CQ), for 30 min. The cells were then further treated with or without 100 nM insulin for additional 8 h. We observed that MG132 treatment alone significantly elevated SESN2 level, while insulin did not further increase SESN2 content in MG132 pretreated cells (Figure 3(b)). In contrast, the lysosomal inhibitor CQ did not exert a notable effect on SESN2 level (Figure 3(b)). Additionally, in the presence of the protein synthesis inhibitor cycloheximide, insulin and MG132 were still able to increase SESN2 content, further supporting our notion that insulin blocks proteasome-mediated SESN2 degradation (Figure 3(c)). If SESN2 is ubiquitinated, we could be able to detect upper bands with higher molecular weights of SESN2 due to its covalent binding to ubiquitins. And, indeed, we observed that both insulin and MG132 treatment enhanced the density of upper bands of SESN2 (Figure 3(d), left panel). Finally, we immunoprecipitated SESN2 protein followed with the polyubiquitin antibody detection and observed that SESN2 protein was polyubiquitinated which was enhanced by both insulin and MG132 (Figure 3(d), right panel).

### 3.4. SESN2 siRNA Knockdown Enhances Insulin Signaling with Reduced PTEN Expression

**Figure 4:**
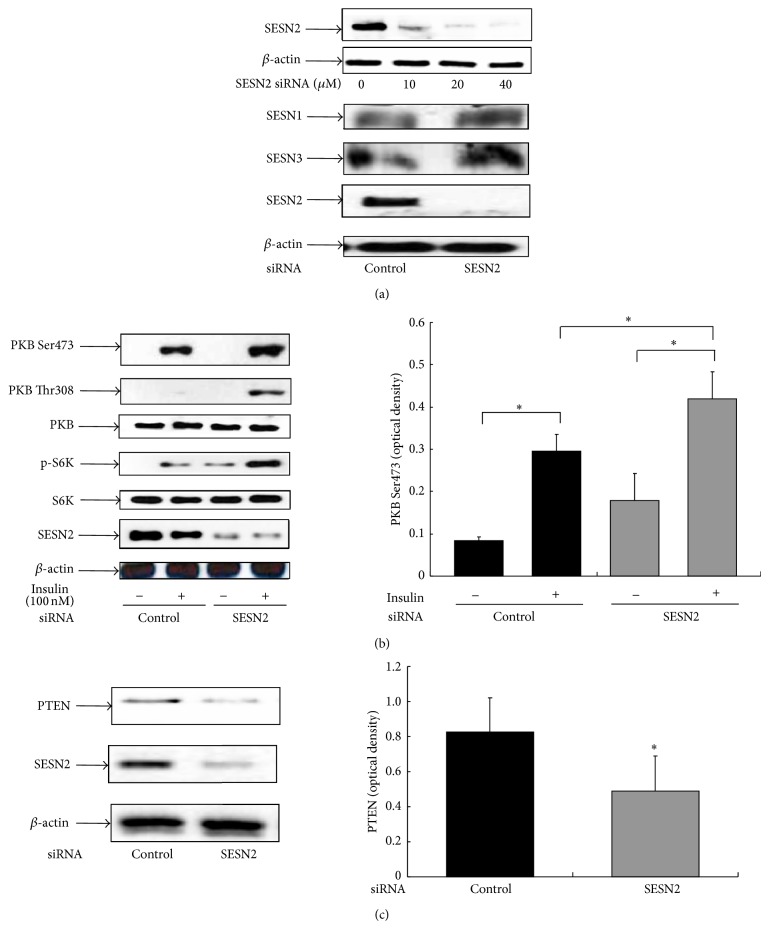
SESN2 siRNA knockdown enhances insulin signaling by repressing the PTEN level. HepG2 cells were transfected with either the control or the SESN2 siRNA for 48–72 h as indicated in Methods. (a) The cells were harvested for western blotting with indicated SESN antibodies. (b) and (c), HepG2 cells were transfected with either the control or the SESN2 siRNA for 48–72 h. The cells were then serum-starved for 4 h, followed by 100 nM insulin treatment for 15 min. Western blotting was performed with indicated antibodies. Right panels show the results of densitometric analysis. ^*^ *P* < 0.05 compared with noninsulin treatment.

Long-term activation of insulin signaling-evoked negative feedback regulation is one of the major mechanisms for downregulating its signaling and this can be achieved by certain downstream signaling components, such as mTOR [18]. To explore a potential contribution of SESN2 in insulin signaling, HepG2 cells were transfected with the SESN2 siRNA or the control siRNA. We observed that SESN2 siRNA concentration dependently reduced SESN2 protein levels (Figure 4(a), upper panel) while other family members of SESN were not affected (Figure 4(a), lower panel). We then examined whether insulin-stimulated PKB phosphorylation could be altered by SESN2 siRNA knockdown. As shown in Figure 4(b), insulin-stimulated PKB phosphorylation at both sites (i.e., Ser473 and Thr308) was enhanced after SESN2 knockdown (Figure 4(b)). In line with this finding, the downstream PKB signaling, S6K phosphorylation was also increased after SESN2 knockdown (Figure 4(b)). Since PKB phosphorylation is regulated by the upstream PI_3_K, we examined the tumor suppressor PTEN, a phosphatase that degrades the PI_3_K product and therefore negatively regulates insulin signaling [18]. As shown in Figure 4(c), SESN2 siRNA knockdown significantly reduced PTEN level to ~50%. These results implicate that insulin-stimulated SESN2 expression will in turn affect its signaling transduction by PTEN-mediated PI_3_K downregulation.

## 4. Discussion

Insulin plays a central role in metabolic regulation and its signaling component mTOR also plays important roles in tumor growth [19]. Hence, understanding the molecular regulatory mechanism of this signaling pathway is essential to further address its etiological role in related diseases. It is well established that insulin signaling-triggered negative feedback is one of basic mechanisms to downregulate insulin function [18]. For example, mTOR and its downstream S6K can inhibit the upstream insulin signaling molecule IRS-1 to regulate glucose uptake and protein synthesis [18, 19]. In the present study, we revealed that insulin by regulating SESN2 protein content via mTOR activation inhibits PKB phosphorylation. These findings provide a novel molecular loop for understanding complicated feedback regulation of insulin signaling network, potentially important for metabolic regulation and tumorigenesis.

In this study we first demonstrated that insulin at physiological concentrations significantly upregulates SESN2 content in both hepatic tumor cell line and mouse primary hepatic cells. This finding is interesting since SESN2 protein expression is elevated in hyperinsulinemia and overactivated mTOR conditions along with its involvement in hepatic metabolic regulations in gluconeogenesis and lipogenesis in vivo [9]. In addition, hyperinsulinemia is linked to several tumorigeneses including hepatic carcinoma [20, 21]. Therefore, it is worth further investigating whether or not insulin could play a role in regulating SESN2 content in vivo and its pathological significance in a variety of metabolic and tumor diseases.

Although, in* Drosophila*, both TOR and JNK activation have been reported to be involved in dSESN upregulation [1], these two signaling pathways in participating mammalian SESN2 expression were not explored prior to our investigation. By employing different inhibitors along insulin signaling pathways, we delineated the critical role of mTOR activation in the upregulation of SESN2 content in HepG2 cells. In contrast, the MAP kinase pathways including JNK appeared not to participate in such an effect of insulin on SESN2 upregulation. Due to incomplete inhibition of chemical inhibitors in MAP kinase activity, we could not completely exclude a role of JNK in SESN2 regulation. However, our data suggest mTOR as a major regulatory signaling molecule in this regulation. We further dissected cellular mechanism responsible for insulin-stimulated SESN2 elevation and several evidences suggest a major role of insulin in blocking SESN2 degradation. First, insulin treatment significantly reduced the disappearance of SESN2 protein in the presence of protein synthesis inhibitor in HepG2 cells, suggesting a modulating effect of insulin on SESN2 turnover. Second, in the presence of the proteasomal inhibitor MG132, insulin treatment did not further increase SESN2 content. And, finally, insulin significantly enhanced SESN2 ubiquitination which was similar to the effect of MG132, indicative of a blockage of SESN2 proteasomal degradation.

Since long-term activation of insulin signaling can evoke an important negative feedback by downstream proteins interacting with the upstream kinases along insulin signaling pathway [18, 20], we explored the potential impact of insulin-stimulated SESN2 expression on insulin signaling transduction. By employing SESN2 siRNA transfection approach in HepG2 cells, we provide the evidence suggesting the existence of an inhibitory function of SESN2 on insulin signaling. This finding is in line with previous reports on the inhibitory feedback role of overactivated mTOR on insulin signaling and extends its molecular loop for this negative regulation [18, 20]. We speculate that this represents a novel mechanism of mTOR by interacting with SESN2 to participate the regulation of insulin signaling. In further support, we found that following SESN2 siRNA transfection in HepG2 cells, PTEN, an important inhibitory phosphatase for PI_3_K activity, was drastically reduced in its protein level. This finding is in compliance with the reported redox regulation in PTEN content [22, 23] and implies that SESN2 knockdown via the alteration of redox balance affects PTEN protein expression. Collectively, these data suggest a regulatory role of SESN2 via changing PTEN content to play a negative feedback function on PI_3_K activity and, therefore, insulin signaling.

A number of studies have addressed the signaling pathways involved in the antitumor function of SESN2. These include the major tumor repressor p53 which is identified as an upstream regulatory protein for SESN2 expression [7]. In addition, SESN2 can interact with other kinases, such as AMPK, to exert its antitumor function by mTOR inhibition [9, 24]. Our present work unveils a potential novel way for the antitumor effect of SESN2 through modulating PTEN and the PI_3_K/PKB signaling. It is interesting that mTOR-stimulated SESN2 activation modulates PTEN, and both mutant PTEN and overactivated mTOR are commonly found in human cancers [19, 21]. Considering that the PI_3_K/PKB/mTOR signaling can be an important machinery for tumor cell survival and anabolic metabolism [20, 21], the functional impact of SESN2 on the regulation of PTEN/PKB signaling in tumor deserves further studies.

Tremendous effect has been made to develop therapeutic reagents for a variety of cancer diseases by targeting mTOR inhibition. The rationale of this strategy is based on the fact that mTOR inhibition will block the downstream oncogenic targets of mTOR signaling proteins including S6K and 4E-BP1 which are critically involved in cancer cell protein synthesis and proliferation [19]. However, to date, the outcome of clinical trials by using mTORC1 inhibitors as a tumor therapeutic approach has not reached a significant success whereas developing mTORC2 inhibitors to enhance therapeutic efficiency is suggested [19]. In addition, mTORC1 inhibition causes other problems, such as insulin resistance and hyperlipidemia [19]. With these challenges in mind, caution is also required to refer to the in vivo significance of present finding.

Together, the present study suggests that insulin could be a physiological regulator of SESN2 protein via mTOR activation, and insulin by blocking SESN2 degradation plays a negative feedback role in insulin signaling transduction.

## Abbreviations

BCA: Bicinchoninic acid
CHX: Cycloheximide
CQ: Chloroquine
DMEM: Dulbecco's Modified Eagle's medium
DTT: Dithiothreitol
MEM: *α*-minimum Eagle's medium
PI_3_K: Phosphoinositide 3-kinase
PKB: Protein kinase B
PMSF: phenylmethylsulphonylfluoride
SDS: Sodium dodecyl sulfate
SESN2: Sestrin 2
TCN: Triciribine.
